# Predictors of Dental Needle Bevel Deformation: Gauge, Insertion Frequency, and Length as Key Determinants

**DOI:** 10.3390/dj14090544

**Published:** 2026-09-01

**Authors:** Osama Zakaria

**Affiliations:** Biomedical Dental Sciences Department, College of Dentistry, Imam Abdulrahman Bin Faisal University, P.O. Box 1982, Dammam 31441, Saudi Arabia; oazakaria@iau.edu.sa

**Keywords:** anaesthesia, dental, needles, equipment failure, patient safety, periodontal ligament, needle deformation

## Abstract

**Background/Objectives:** Dental needles are used routinely for local anaesthesia, but repeated insertion, needle selection, and operator handling may all compromise tip integrity. This study assessed bevel deformation, biological debris, and manual bending in used dental needles collected during undergraduate clinical practice. Their associations with needle dimensions, insertion frequency, injection technique, and operator seniority were also examined. **Methods:** 192 discarded needles were collected from fourth-, fifth-, and sixth-year students and dental interns after routine local anaesthesia procedures at an academic dental hospital. Each needle was examined under stereomicroscopy. Needle bevels were classified as intact, outward bending, or inward bending; biological debris and manual bending were also recorded. Predictors of bevel deformation were identified by multivariable logistic regression. **Results**: Bevel deformation was present in 93 of 192 needles. Thirty-gauge needles showed higher odds of deformation than 27-gauge needles (OR = 2.54, *p* = 0.004), and each additional insertion further increased the odds (OR = 1.98, *p* = 0.016); once gauge was accounted for, needle length was not an independent predictor. Intended bone contact showed no association with deformation. Outward bending predominated among deformed tips (63.4% vs. 36.6% inward), and biological debris was detected on 16.1% of needles. Manual bending was more frequent among interns than students (OR = 3.67, *p* = 0.007). **Conclusions:** Used dental needles frequently exhibited bevel deformation, particularly with 30-gauge needles and after repeated insertions. The occurrence of deformation even in the absence of intentional bone contact further supports strict single-use practices and careful needle handling during local anaesthesia. Although the higher frequency of manual needle bending observed among interns should be interpreted cautiously, this finding highlights a potential area for targeted operator training and further investigation.

## 1. Introduction

Needle deformation and fracture are recognised mechanical complications of dental local anaesthesia. Although fracture is now rare, owing to modern manufacturing and disposable single-use needles, it remains a serious event that can cause patient morbidity, injury to vital structures, and litigation [1,2]. When breakage occurs it most often involves the inferior alveolar nerve block and is typically attributable to preventable factors such as inadequate technique, the use of thin (30-gauge) needles, or insertion to the hub [3]. Manual bending of the shaft for easier access is a particularly relevant antecedent, as it introduces stress risers that predispose the needle to failure; bending is nonetheless common, with a substantial proportion of clinicians acknowledging the practice, and is considered acceptable only for specific techniques rather than as a routine manoeuvre [4,5].

Even when a procedure does not require osseous contact, needle-tip damage is well documented. Rout et al. [6] reported that 25% of needles were deformed after a single injection with no apparent bone contact, rising to 97.3% when bone was contacted; 17% also retained soft-tissue or blood debris. Stacy and Hajjar [7] found that 60% of needles used for mandibular blocks were barbed; when these barbs face outward, they may tear tissue, nerves, and blood vessels as the needle is withdrawn.

Notably, such damage is not confined to clinical use. Gaitán-Fonseca et al. [8] examined unused 30-gauge needles by scanning electron microscopy at 900× magnification and found the bevel tips already deformed by −4.06° to −9.12° relative to the horizontal plane. 

This deviation, present before clinical use and invisible to the naked eye, underscores that even new needles may not fully meet the dimensional and mechanical specifications set for needle tubing (ISO 9626) [9]. Deformation is most pronounced in high-pressure techniques. These considerations are particularly relevant to endodontic practice, where periodontal-ligament (intraligamentary) injections are frequently used as primary or supplemental anaesthesia, often under high injection pressure, so that needle-tip integrity directly influences both the safety and effectiveness of the technique. Kämmerer et al. [10] reported deformation in 96–100% of needles used for intraligamentary anaesthesia, and Kuzin et al. [11] observed severe “U-shaped” bends in 77.8% of regular-bevel needles used for PDL injection. Bevel geometry, in turn, governs the mechanical behaviour of the needle: Steele et al. [12] showed in vitro that bevel design significantly affects the forces required to penetrate and withdraw the needle, and Dau et al. [13] showed that bevel design influences tip deformation during local infiltration.

Needle gauge intensifies these mechanical vulnerabilities. Thirty-gauge needles are often preferred to reduce pain on insertion, but they are susceptible to deflection and fracture when compared with 27-gauge variants [14].

Despite this body of evidence, the morphology of deformation in used dental needles, and its relationship to needle dimensions, injection technique, and operator factors, has not been comprehensively characterised within a single clinical sample.

The present study therefore aims to characterise the morphology and mechanical correlates of used-needle deformation within a university dental hospital setting, describing its association with needle dimensions, clinical technique, and operator seniority.

## 2. Materials and Methods

### 2.1. Study Design and Ethical Considerations

This was a cross-sectional morphological audit of discarded dental needles. The protocol was approved by the Institutional Review Board of the hosting institution (IRB-2024-02-753) and conducted in accordance with the ethical standards of the 1964 Declaration of Helsinki. Needles were collected consecutively between November 2024 and October 2025. The unit of analysis was the individual discarded needle. Operators were not individually identified—no name, student code, or chair number was recorded—so multiple needles from the same operator could not be linked, and the number of distinct operators contributing to each grade is unknown.

### 2.2. Sample Collection and Operator Characteristics

A total of 192 disposable dental needles were collected from the university dental clinics following routine local anaesthesia procedures. Operators comprised fourth-, fifth-, and sixth-year undergraduate dental students and dental interns. For each needle, the operator was asked at the point of collection which dental procedure had been performed and which anaesthetic technique(s) had been administered with that needle; the operator’s academic grade and sex, the reported technique(s), and the procedure were recorded on a standardised data-collection form (Supplementary Materials), and the needle was stored in a pouch bearing a serial number matching the form entry.

Insertion frequency was derived from this record as the number of distinct techniques reported per needle, on the assumption that each technique represented a separate insertion; this does not capture repeated insertions within a single technique (e.g., re-insertion after a failed block), nor exclude the possibility that a single insertion occasionally served more than one target.

Two needles were recorded as 30-gauge but had a 0.4 mm (27-gauge) width; they were retained as recorded in the descriptive cross-tabulation (Table 1) but excluded from the gauge-dependent regression analyses, leaving 190 needles for those analyses.

The sample of 192 needles was considered adequate to detect clinically meaningful differences in deformation frequency, given that previously reported deformation rates range widely, from approximately 25% in the absence of bone contact [6] to 100% following repeated needle use [15].

### 2.3. Needle Specifications

All needles were disposable tri-bevel C-K Ject Premium dental injection needles (C-K Dental Ind. Co., Ltd., Bucheon-si, Republic of Korea). In the collected sample, 27-gauge needles were present at 21, 25, and 30 mm lengths and 30-gauge needles at 25 mm. All injections were delivered using Septodont anaesthetic cartridge 2% lidocaine with 1:100,000 epinephrine. (Septodont, Saint-Maur-des-Fossés, France).

### 2.4. Imaging and Morphological Audit

All 192 needles were imaged under standardised stereomicroscopy at fixed magnification and illumination using a LUXO binocular stereomicroscope (Luxo, Elmsford, NY, USA) fitted with a 1.0× objective and 10× eyepieces, at a working magnification of approximately 7× to 45×, providing a complete photographic record for every specimen. This image archive formed the basis for the morphological classification and for the reliability assessment described below. To distinguish clinical damage from manufacturing defects, a control set of 30 unused needles (15 per gauge) was examined under identical conditions to establish baseline tip integrity.

**Figure 1:**
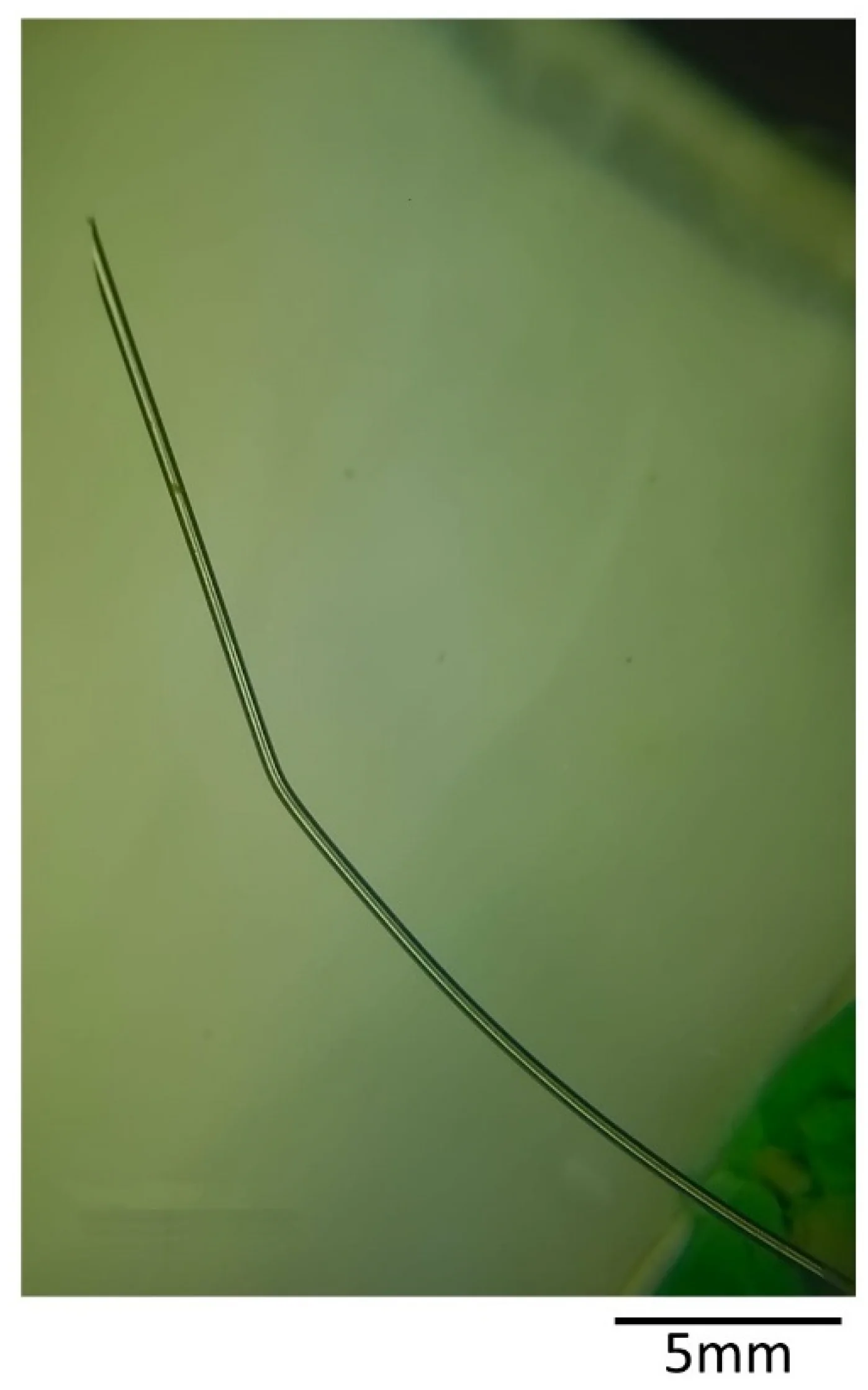
Stereomicrograph of a manually bent 27-gauge dental needle, showing an angular deflection of the shaft consistent with intentional bending for access. Manual bending was recorded on 21 needles (10.9%), most frequently in the hub region (61.9%). Scale bar = 5 mm.

A systematic morphological audit evaluated bevel integrity and directionality, shaft integrity, biological debris, and safety compliance, using the following operational definitions:

Manual bending: intentional deformation of the needle shaft by the operator, with the location recorded (hub region, mid-shaft, near bevel, or multiple sites (Figure 1).

**Figure 2:**
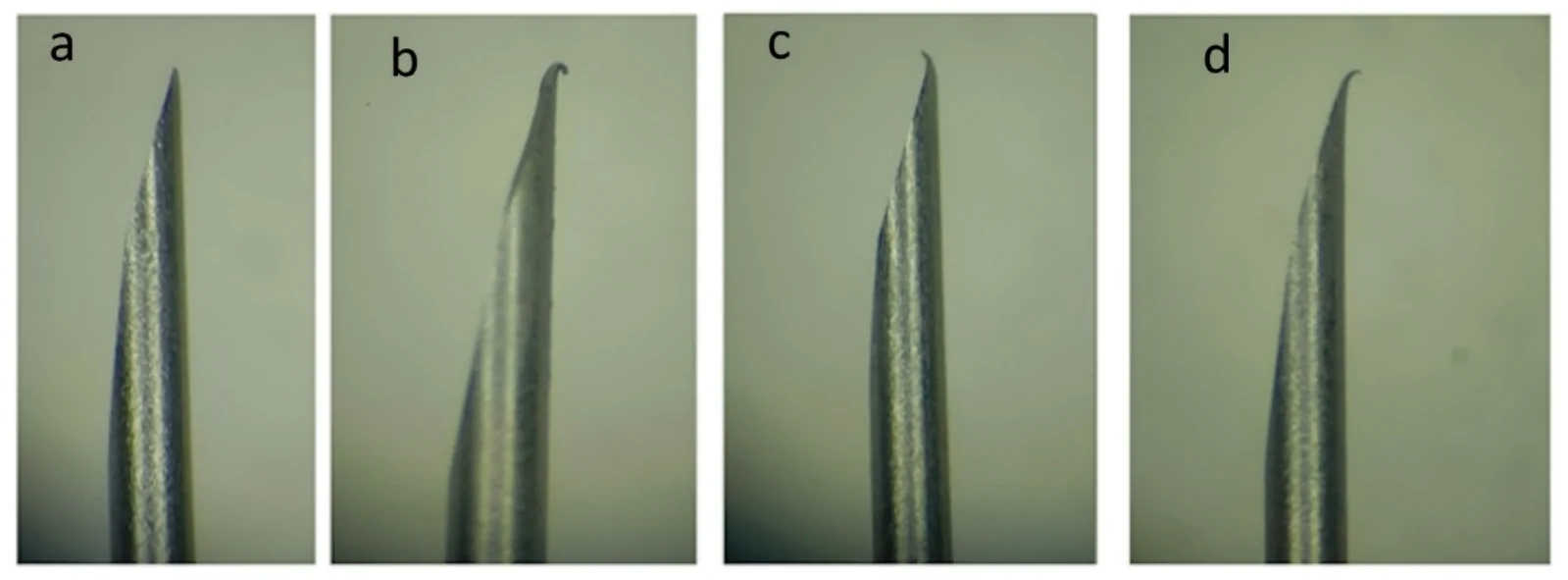
Stereomicroscopic appearance of dental needle tips. (**a**) Intact bevel with standard tri-bevel point geometry, as observed in unused control needles. (**b**) Severe “fishhook” barbing, with the tip curled into a hook that creates marked mechanical resistance on withdrawal. (**c**) “Inside” bending, with the bevel deformed toward the lumen. (**d**) ‘Outside’ bending: bevel curl directed away from the lumen. Because intended bone contact was not associated with deformation, such damage is interpreted as arising from inadvertent periosteal contact rather than from deliberate osseous contact. Scale bar = 0.3 mm.

Tip deformation (barbing): unintended bevel damage classified as “outside bending” (curl away from the lumen) or “inside bending” (curl toward the lumen) (Figure 2).

**Figure 3:**
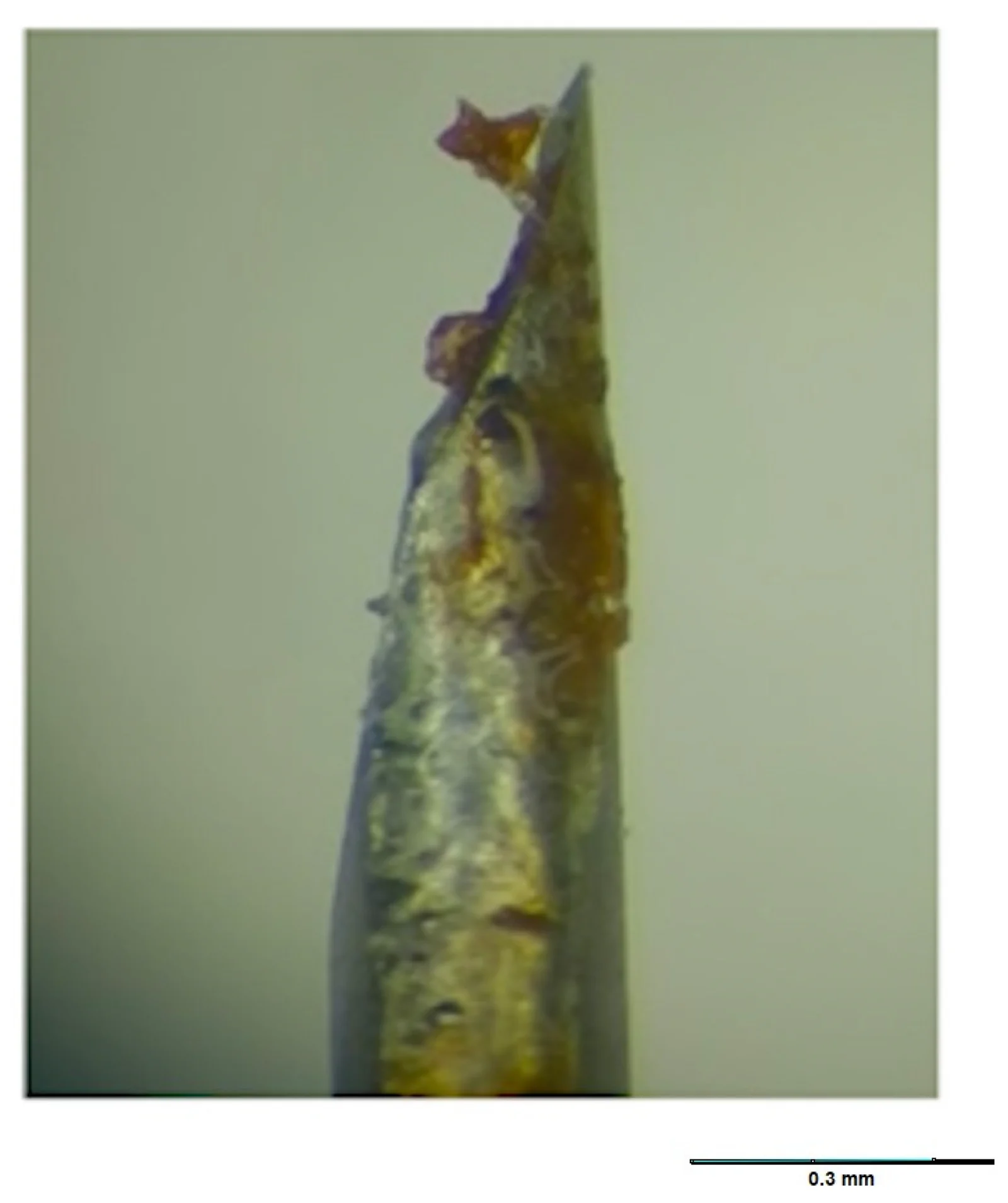
Biological debris retained on a deformed needle tip. Tissue and blood aggregates are visible within the barbed tip, illustrating the “fishhook” mechanism by which microscopic deformation facilitates retention and transport of biological material on withdrawal; debris was detected on 16.1% of needles. Scale bar = 0.3 mm.

Biological debris: detection of tissue or blood aggregates by stereomicroscopy, taken as a marker of the “fishhook” effect on withdrawal (Figure 3).

Safety compliance: presence of manual bending and failure to recap the cartridge-penetrating end.

### 2.5. Examiner Calibration and Reliability

Each needle was examined under stereomicroscopy by a single calibrated examiner. Bevel condition was classified as intact, outside bending (curl away from the lumen), or inside bending (curl toward the lumen); biological debris and manual bending were recorded as present or absent, with the location of any manual bend noted (hub region, mid-shaft, near bevel, or multiple sites). To assess reproducibility, a second calibrated examiner independently re-scored a random 20% subset (n = 38), blinded to operator grade and technique; inter-examiner agreement for the ordinal bevel classification was almost perfect (weighted Cohen’s κ = 0.81).

### 2.6. Statistical Analysis

Data were analysed using Python (v3.12) with the statsmodels package (v0.14.6). Categorical variables were summarised as frequencies and percentages. Insertion frequency was not recorded directly; it was derived for each needle as the number of distinct anaesthetic techniques documented on the data-collection form, assuming a single insertion per technique. Repeated insertions within the same technique were therefore not captured. Multivariable logistic regression was used to identify predictors of needle bevel deformation (categorised as either deformed or intact). This model included the following predictors simultaneously including needle gauge, needle length, frequency of insertion, and the operator academic level (intern vs. student). The predictors of deliberate manual bending (21 occasions) were analysed separately, using univariable logistic regression as operator academic level and periodontal-ligament (PDL) injection, due to the rarity of this event. Fisher’s exact test was used for the small PDL subgroup. These associations are reported as odds ratios (OR) with 95% confidence intervals (CI). Model reliability was confirmed using the Hosmer–Lemeshow goodness-of-fit test (χ^2^ = 1.72, df = 5, *p* = 0.887), and multicollinearity was ruled out since all variance inflation factors were under 1.2. Two needles missing dimensional or gauge data were excluded from any gauge-specific analyses, and the threshold for statistical significance was set at a two-sided *p*-value < 0.05. No a priori sample-size calculation was performed; all needles discarded during the study period were analysed (a convenience sample). Post hoc, this sample provided adequate precision: for a proportion of approximately 50%, the 95% confidence half-width is about ±7%, and the study had greater than 90% power at α = 0.05 to detect the observed between-gauge difference in deformation (≈26 percentage points). To limit overfitting, the deformation model included four predictors against 93 events (≈23 events per predictor), and the less frequent manual-bending outcome (21 events) was analysed by univariable logistic regression and Fisher’s exact test rather than a multivariable model.

## 3. Results

### 3.1. Sample Characteristics

A total of 192 dental needles were analysed, with an equal operator-sex distribution (96 female, 96 male).The operators included fourth-year (n = 44, 22.9%), fifth-year (n = 49, 25.5%), and sixth-year (n = 61, 31.8%) undergraduate students, alongside dental interns (n = 38, 19.8%). In terms of needle thickness, 101 were 27-gauge and 89 were 30-gauge; two needles were excluded from gauge-specific analyses due to missing data. Based on the recorded clinical sequences, 102 needles (53.1%) were used for a single insertion, 83 (43.2%) for two insertions, and 7 (3.6%) for three.

### 3.2. Needle Deformation and Morphology

Control analysis of 30 unused needles (15 of each gauge) under stereomicroscopy revealed zero visible bevel deformation, ruling out manufacturing defects in the sampled batches. However, among the clinically used needles, 93 out of 192 (48.4%) exhibited bevel deformation. This damage was significantly more common in 30-gauge needles than in 27-gauge needles (62.9% vs. 36.6%). Among the 93 deformed tips, outward bending (curling away from the lumen) was the dominant pattern, accounting for 59 cases (63.4%), while inward bending accounted for 34 cases (36.6%) (Figure 2). Notably, deformation occurred in techniques not requiring osseous contact as often as in those that do (47.4% vs. 49.1%; Fisher’s exact test, *p* = 0.88), indicating that intended bone contact was not associated with deformation and implicating frequent inadvertent periosteal contact.

### 3.3. Multivariable Correlates of Deformation

In a multivariable logistic regression model (Table 2, Section A), needle gauge and insertion frequency independently predicted bevel deformation. Thirty-gauge needles carried more than twice the odds of deformation compared with 27-gauge variants (OR = 2.54, 95% CI 1.34–4.82, *p* = 0.004); each additional insertion of the same needle increased the odds of deformation (OR = 1.98, 95% CI 1.14–3.46, *p* = 0.016); In the pooled model, greater needle length appeared protective (OR = 0.90 per mm, 95% CI 0.81–0.99, *p* = 0.034); however, because 30-gauge needles were of a single length (25 mm) and length varied almost exclusively within the 27-gauge stratum (Table 2), this effect could not be separated from gauge, and within the 27-gauge subgroup alone it was non-significant (OR ≈ 0.92 per mm, 95% CI 0.83–1.02, *p* = 0.12). Needle length is therefore not interpreted as an independent determinant of deformation. The model showed good calibration (Hosmer–Lemeshow *p* = 0.887) with no evidence of multicollinearity (all variance inflation factors < 1.2; gauge–length r = −0.36).

### 3.4. Biological Debris

Biological debris was identified on 31 needles (16.1%) (Figure 3). Debris was not significantly more frequent on deformed than on intact needles (OR = 1.36, Fisher *p* = 0.56), and no significant association was found with operator sex, grade, or injection technique (all *p* > 0.05).

### 3.5. Operator Seniority (Secondary Findings)

**Figure 4:**
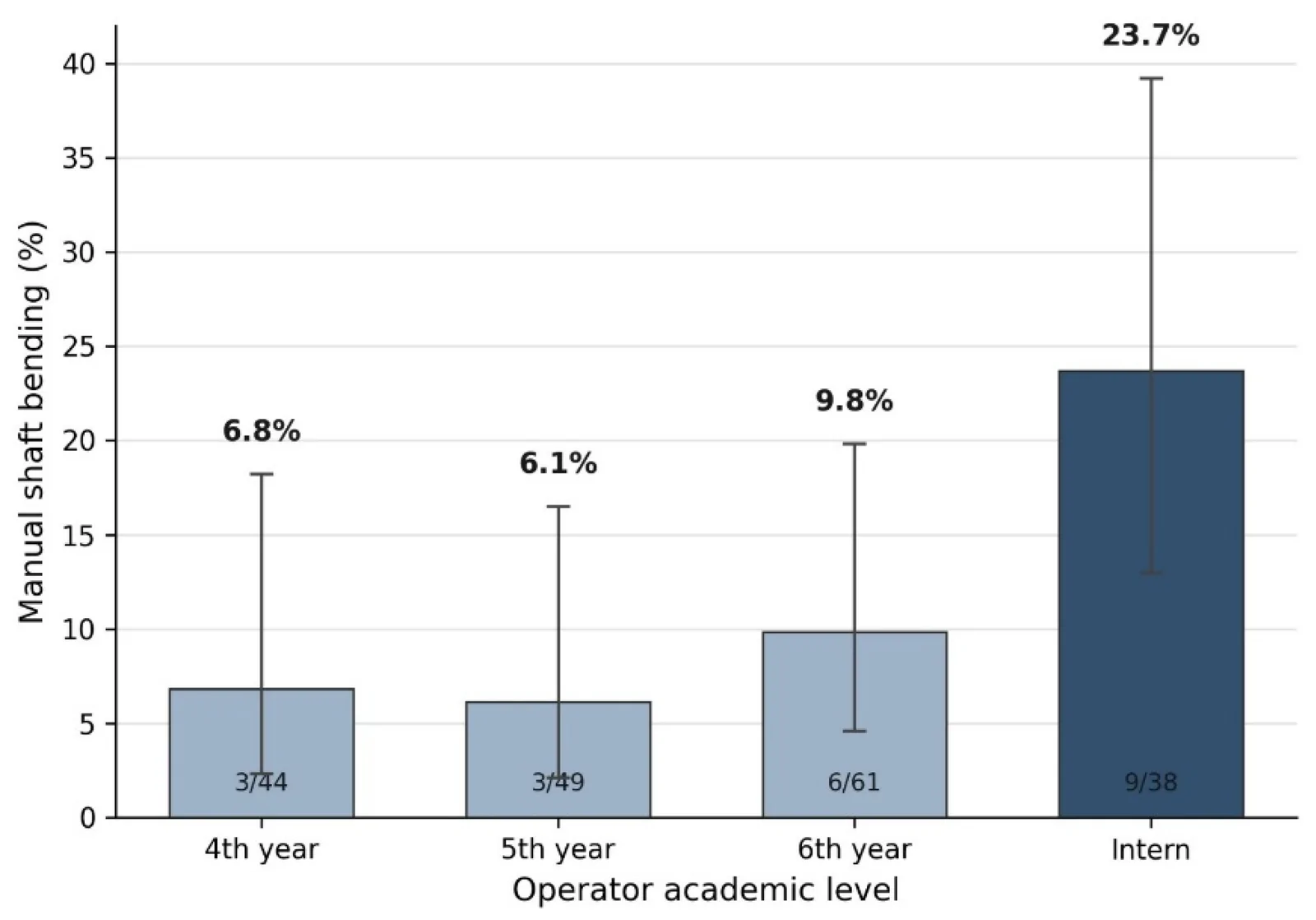
Prevalence of intentional manual bending by operator academic level. Bars show the percentage of returned needles exhibiting manual bending within each grade; error bars are 95% Wilson confidence intervals, and fractions denote bent/total needles per grade. Bending was infrequent and similar across the student years (4th–6th) and higher among interns. Because operators were not individually identified, the intern estimate may reflect the handling of a small number of operators and should be interpreted as a hypothesis-generating signal.

Operator seniority was examined as a descriptive covariate. Deformation was more frequent among needles returned by interns (63.2%) than students (44.8%), but this association did not persist after adjustment for needle gauge, insertion frequency, and length (adjusted OR = 1.93, 95% CI 0.88–4.19, *p* = 0.099), suggesting it is largely explained by needle-related factors rather than seniority per se. By contrast, intentional manual bending—recorded on 21 needles (10.9%) overall—was clearly more frequent among interns (23.7%) than students (7.8%) (OR = 3.67, 95% CI 1.42–9.52, *p* = 0.007) (Figure 4). Bend location was recorded for all affected needles: of the 21 needles showing manual bending, 13 (61.9%) were bent in the hub region, 4 (19.0%) at the mid-shaft, 2 (9.5%) near the bevel, and 2 (9.5%) at multiple sites. Manual bending was most strongly associated with periodontal-ligament (PDL) injection, where 5 of 8 needles (62.5%) were bent; however, this rests on a very small subgroup and was examined by Fisher’s exact test (OR ≈ 17.5, *p* < 0.001). Because operators were not individually identified, multiple needles from the same operator could not be linked, and these seniority- and technique-related associations may reflect the handling of a small number of operators rather than a grade-wide effect. They are therefore presented as hypothesis-generating signals warranting prospective, operator-level study, rather than confirmed behavioural effects.

## 4. Discussion

This morphological audit characterised deformation in used dental needles and its relationship to needle dimensions, injection technique, and operator seniority. The principal findings were mechanical. Deformation was common and was driven chiefly by needle gauge and by repeated insertion, occurring even in techniques not intended to contact bone.

### 4.1. Gauge, Insertion, and Mechanical Vulnerability

The doubled odds of deformation in 30-gauge compared with 27-gauge needles (OR = 2.54) are consistent with the accepted trade-off between the decreased insertion pain of thinner needles and their increased susceptibility to deflection and fracture [14]. This vulnerability was most evident in PDL injection, which also showed the greatest odds of manual bending. This finding supports Malamed’s [5] recommendation to use 27-gauge needles for high-pressure intraligamentary techniques and is in line with the findings of Kuzin et al. [11], who documented severe tip damage in almost 78% of needles used for intraligamentary anaesthesia. The odds of deformation increasing with each subsequent insertion (OR = 1.98) support the use of single-use needle protocols, as repeated penetration progressively damages the needle tip and increases the risk of iatrogenic trauma [16]. These findings are also pertinent to contemporary conservative and regenerative endodontic care, where the preservation of vital tissue and atraumatic technique are emphasised, and where supplemental intraligamentary anaesthesia is commonly required [17].

### 4.2. Deformation in Non-Contact Techniques and Bending Directionality

Bevel damage observed in techniques where osseous contact is not intended, such as posterior superior alveolar (PSA) and infiltration blocks, along with the absence of any association between intended bone contact and deformation (Fisher’s exact *p* = 0.88), suggests frequent inadvertent periosteal contact.

This pattern is consistent with Rout et al. [6], who observed tip deformity even without apparent bone contact. Almendros-Marqués et al. [18] attributed such distortion to mechanical factors—chiefly excessive insertion force rather than deliberate osseous contact. The direction of deformation carries clinical weight. Outside bending predominated (63.4%), and outward-facing barbs pose the greatest risk of tearing tissue, nerves, and vessels on withdrawal [7]. The control needles showed no visible deformation, in contrast to Gaitán-Fonseca et al. [8], who detected microscopic bevel damage in new needles at 900×. This discrepancy most plausibly reflects magnification: at the lower power used here, subtle sub-threshold defects may remain undetected.

### 4.3. Biological Debris and Tissue Trauma

Debris appeared on 16.1% of needles. This is close to the 17% Rout et al. [6] found. A hooked tip can trap tissue or blood in the crook of the barb, and when the needle is withdrawn, that material may be transported deeper into the tissues. The finding is a reminder that even soft-tissue injections can leave microscopic barbs [7]. This provides a further mechanical rationale for single-use protocols.

### 4.4. Operator Seniority: A Hedged Observation

Needles returned by interns more often showed manual bending (OR = 3.67); the higher frequency of physical deformation among interns, however, did not persist after adjustment for needle-related factors (adjusted OR = 1.93, *p* = 0.099), and manual bending was most strongly associated with PDL injection (5 of 8 needles; Fisher’s exact OR ≈ 17.5). The concentration of bending in the hub region (61.9%) is consistent with deliberate manipulation of the needle to improve access, rather than incidental deformation during insertion. These observations are consistent with a proposed pattern in which intentional shaft manipulation becomes more frequent with clinical seniority. This pattern must, however, be interpreted with caution. Operators were not individually identified, so multiple needles from the same operator could not be linked; the seniority associations may therefore reflect the handling of a small number of operators rather than a grade-wide effect. The estimates for manual bending—particularly the wide confidence interval around the intern OR and the very small PDL subgroup—are correspondingly fragile. We therefore present this seniority pattern as a hypothesis-generating signal rather than a confirmed behavioural effect, warranting prospective, operator-level investigation. Behavioural and educational interventions targeting needle handling lie beyond the scope of this morphological study.

### 4.5. Limitations

Several limitations should be acknowledged. First and most importantly, because operators were not individually identified, needles from the same operator could not be linked and were treated as independent; this clustering could not be modelled or corrected retrospectively, and mostly affects the analyses of operator seniority, manual bending, and injection technique, where the estimates could be disproportionately influenced by a small number of high-volume operators. This is the principal reason these findings are presented as exploratory rather than confirmed. Second, the cross-sectional design precludes causal inference and cannot establish a developmental trajectory of handling behaviour. Third, insertion frequency was a recorded proxy—the number of operator-reported techniques per needle—rather than a directly observed count; it could not capture repeated insertions within a single technique (e.g., re-insertion after a failed block), and this misclassification, likely non-differential with respect to deformation, would be expected to bias the association toward the null, so the true effect may be underestimated. Finally, the study used a convenience sample rather than one sized by a formal a priori calculation, and power for the less frequent behavioural outcomes was correspondingly limited.

## 5. Conclusions

Within these limitations, used dental needles frequently sustained bevel deformation that was more likely with 30-gauge needles and with repeated insertions, and that occurred even in techniques not intended to contact bone. These findings support single-use needle protocols, selection of 27-gauge needles for high-pressure techniques such as PDL injection, and heightened awareness of inadvertent periosteal contact during infiltration. The greater frequency of manual bending among interns is an exploratory, hypothesis-generating observation that warrants prospective, operator-level study before any inference about clinical seniority can be drawn.

## Figures and Tables

**Table 1:** Cross-tabulation of needle gauge and length in the analysed sample (n = 190).

Gauge	21 mm	25 mm	30 mm	Total
27G	28	5	68	101
30G	0	87	2	89
Total	28	92	70	190

Two needles recorded as 30-gauge had a 0.4 mm (27-gauge) width; they are shown here as recorded but were excluded from gauge-dependent regression analyses. Length varied almost exclusively within the 27-gauge stratum.

**Table 2:** Predictors of needle deformation and manual bending, and descriptive morphological characteristics (n = 192).

Category	Variable	Findings	OR (95% CI)	*p*
Section A. Predictors of bevel deformation (multivariable logistic regression)				
Needle gauge	30G vs. 27G	62.9% vs. 36.6%	2.54 (1.34–4.82)	0.004 *
Insertion frequency	Per additional insertion	—	1.98 (1.14–3.46)	0.016 *
Needle length ^a^	Per 1 mm increase	—	0.90 (0.81–0.99)	0.034 *
Academic level	Interns vs. students	63.2% vs. 44.8%	1.93 (0.88–4.19)	0.099
Section B. Predictors of intentional manual bending				
Academic level	Interns vs. students	23.7% vs. 7.8%	3.67 (1.42–9.52)	0.007 *
PDL injection ^b^	PDL vs. other techniques	5/8 (62.5%) bent	17.50 (Fisher)	<0.001 *
Section C. Descriptive morphological characteristics				
Bend directionality	Outside vs. inside	63.4%/36.6% (n = 59/34)	—	—
Biological debris	Incidence	16.1% (n = 31)	—	—

Data are frequencies, percentages, or odds ratios (OR) with 95% confidence intervals (CI). * *p* < 0.05. Gauge-dependent analyses were based on 190 needles (two needles with a gauge/width discrepancy excluded). The Section A model included needle gauge, insertion frequency, needle length, and operator academic level; injection technique and intended bone contact were examined as bivariate associations only. Model calibration was adequate (Hosmer–Lemeshow *p* = 0.887; all variance inflation factors < 1.2). Academic level, technique, and seniority associations are descriptive: operators were not individually identified, so needles could not be linked to individual operators. ^a^ Pooled model only; needle length was not an independent predictor—it varied almost exclusively within the 27-gauge stratum and was non-significant within that subgroup (OR ≈ 0.92 per mm, *p* = 0.12). ^b^ Predictors of manual bending were assessed individually owing to the small number of events (n = 21); the PDL estimate is based on 8 needles and is statistically imprecise (Fisher’s exact test). Academic level, technique, and seniority associations should be interpreted as descriptive: operators were not individually identified, so needles could not be linked to individual operators.

## Data Availability

The dataset and the morphological image archive (all 192 needles) generated and analysed in this study are available from the corresponding author on reasonable request.

## Supplementary Materials

The following supporting information can be downloaded at: https://www.mdpi.com/article/10.3390/dj14090544/s1.

## Abbreviations

The following abbreviations are used in this manuscript:

CI	confidence interval
IRB	Institutional Review Board
OR	odds ratio
PDL	periodontal ligament
PSA	posterior superior alveolar
VIF	variance inflation factor
